# The LH–DH module of bacterial replicative helicases is the common binding site for DciA and other helicase loaders

**DOI:** 10.1107/S2059798323000281

**Published:** 2023-02-06

**Authors:** Claire Cargemel, Stéphanie Marsin, Magali Noiray, Pierre Legrand, Halil Bounoua, Inès Li de la Sierra-Gallay, Hélène Walbott, Sophie Quevillon-Cheruel

**Affiliations:** a Université Paris-Saclay, CEA, CNRS, Institute for Integrative Biology of the Cell (I2BC), 91180 Gif-sur-Yvette, France; b Synchrotron SOLEIL, L’Orme des Merisiers, 91192 Gif-sur-Yvette, France; University of Queensland, Australia

**Keywords:** DnaB·DciA complex, crystal structure, helicase loaders, bacterial replicative helicases, interaction modules, replicative helicase hijacking, *Vibrio cholerae*

## Abstract

During the initiation step of bacterial genome replication, replicative helicases depend on specialized proteins for their loading onto *oriC*. DnaC and DnaI were the first loaders to be characterized. However, most bacteria do not contain any of these genes, which are domesticated phage elements that have replaced the ancestral and unrelated loader gene *dciA* several times during evolution. To understand how DciA assists the loading of DnaB, the crystal structure of the complex from *Vibrio cholerae* was determined, in which two *Vc*DciA molecules interact with a dimer of *Vc*DnaB without changing its canonical structure. The data showed that the *Vc*DciA binding site on *Vc*DnaB is the conserved module formed by the linker helix LH of one monomer and the determinant helix DH of the second monomer. Interestingly, DnaC from *Escherichia coli* also targets this module onto *Ec*DnaB. Thanks to their common target site, it was shown that *Vc*DciA and *Ec*DnaC could be functionally interchanged *in vitro* despite sharing no structural similarity. This represents a milestone in understanding the mechanism employed by phage helicase loaders to hijack bacterial replicative helicases during evolution.

## Abstract 2

The crystallographic structure of the DnaB·DciA complex from *Vibrio cholerae* reveals that the various helicase loaders share the same binding site on the bacterial replicative helicase, albeit with differences in their loading mechanisms.

## 1. Introduction

The replication of the circular bacterial chromosome is an essential step for bacterial division. The initiator protein DnaA initiates replication by binding onto the origin DNA *oriC* and locally unwinds the double-stranded DNA by polymerization (Costa *et al.*, 2013 ▸; Leonard & Méchali, 2013 ▸; Zawilak-Pawlik *et al.*, 2017 ▸). The toroidal hexameric helicase DnaB is then loaded onto the locally open DNA duplex, with the help of a helicase loader, triggering the recruitment of the various proteins of the replisome (O’Donnell *et al.*, 2013 ▸). ATP-dependent 5′-to-3′ translocation of the helicase ahead of the advancing replisome allows the unwinding of the DNA duplex into templates for new DNA synthesis (Strycharska *et al.*, 2013 ▸).

Recruitment and loading of the replicative helicase depend on a loader protein, which has been characterized in the two model organisms *Escherichia coli* (*Ec*) and *Bacillus subtilis* (*Bs*), leading to the description of two loading strategies (see Table 1 ▸). In *B. subtilis*, the helicase loader DnaI assists the assembly of six monomers of the helicase to form an active hexameric ring around DNA according to a ‘ring-maker’ scenario (Davey & O’Donnell, 2003 ▸; Velten *et al.*, 2003 ▸). The *Gst*DnaB·*Bs*DnaI·*Gst*DnaG prepriming complex exhibits a three-layered planar and dilated ring conformation with one hexameric helicase binding to three loader-protein dimers and three primase proteins (Liu *et al.*, 2013 ▸). In the *E. coli* system, the helicase loader DnaC mediates the opening of the DnaB hexamer into a loading-competent cracked open ring according to a ‘ring-breaker’ scenario (Arias-Palomo *et al.*, 2019 ▸; Nagata *et al.*, 2020 ▸). The *Ec*DnaB·*Ec*DnaC complex is dodecameric, with six subunits of each protein, and the complex assembles into a three-tier spiral. *Ec*DnaC makes contact with *Ec*DnaB through the first small α-helix (15 residues in length) of its extended N-terminal domain (NTD). This helix interacts with a DnaB module composed of the ‘linker helix’ (LH) of one DnaB protomer and an antiparallel α-helix of the adjacent DnaB protomer, which we named the ‘determinant helix’ (DH) in a previous study (Marsin *et al.*, 2021 ▸) and which was named the ‘docking helix’ in a recent review comparing the convergent functional mechanisms of *Ec*DnaC and bacteriophage λ P loaders (Chase *et al.*, 2022 ▸). This forms a three α-helix bundle which fixes the relative orientation of the two adjacent DnaB C-terminal domains (CTDs): the six DnaC molecules thus latched onto the DnaB hexamer adopt a spiral configuration that causes distortion of the helicase ring, resulting in its large opening as a means to allow single-strand DNA (ssDNA) to enter the helicase pore (Arias-Palomo *et al.*, 2019 ▸; Nagata *et al.*, 2020 ▸).

Despite this, the DnaC/I loader distribution is marginal in the bacterial domain. It was established phylogenetically that the *dnaC*/*I* genes are domesticated phage elements that have replaced the ancestral bacterial gene *dciA* several times during evolution (Brézellec *et al.*, 2016 ▸). DciA and DnaC/I are not related in either their sequence or their structure (Marsin *et al.*, 2021 ▸). While the DnaC/I CTD contains an AAA+ ATPase RecA-like domain (Koonin, 1992 ▸), the DciA NTD folds as a KH domain (Grishin, 2001 ▸; Marsin *et al.*, 2021 ▸), also shared by domain I of DnaA and domain V of DnaX, which have both been described to interact with DnaB (Rajathei & Selvaraj, 2013 ▸; Jameson *et al.*, 2014 ▸; Haroniti *et al.*, 2004 ▸; Mann *et al.*, 2017 ▸). Through multiple complementary approaches, we previously established that the disordered CTD of DciA can form transiently small helical structures (Chan-Yao-Chong *et al.*, 2020 ▸) and is necessary for interacting with DnaB and stimulating the loading of DnaB onto DNA (Chan-Yao-Chong *et al.*, 2020 ▸; Marsin *et al.*, 2021 ▸). Direct interplay between the two proteins has also been demonstrated using the variation of intrinsic fluorescence of the conserved tryptophan residue located in the middle of the DH of DnaB in the presence of DciA (Marsin *et al.*, 2021 ▸). It was therefore suspected that DciA interacts with the helicase near to the DH helix of its module.

To decipher the molecular interactions between DciA and DnaB, we solved the crystal structure of the DnaB·DciA complex from *Vibrio cholerae* (*Vc*) together with ADP and Mg^2+^, forming a heterotetramer composed of the canonical *Vc*DnaB dimer and two molecules of *Vc*DciA. Interestingly, *Vc*DciA interacts with *Vc*DnaB through the LH–DH module, like *Ec*DnaC and the phage λ P helicase loader on *Ec*DnaB, suggesting a functional link between the different systems. Furthermore, we showed that *Vc*DciA and *Ec*DnaC are interchangeable for *in vitro* loading of the helicases from *V. cholerae* and *E. coli*, suggesting convergent evolution of both helicase-loader systems. However, the *Vc*DnaB·*Vc*DciA·ADP:Mg^2+^ structure also revealed that DciA binds to the periphery of the helicase CTD, in contrast to other known loaders that oligomerize and are positioned at the back of the DnaB CTD ring, leading to the presumption that its helicase-loading mechanism differs from those of DnaC/I and λ P.

## 2. Materials and methods

### 2.1. Protein-sample preparation

*Vc*DnaB, *Ec*DnaB, *Vc*DciA, *Vc*DciA^(1–111)^ and *Ec*DnaC were overexpressed in *E. coli* and purified as described in Marsin *et al.* (2021 ▸). *Ec*DnaC^(53-end)^ was purified in the same way as *Ec*DnaC. Strains and plasmids are available upon request.

### 2.2. Crystal structure determination of the *Vc*DnaB·*Vc*DciA·ADP:Mg^2+^ complex

Purified *Vc*DnaB was pre-incubated for 10 min at 4°C at a concentration of 0.115 m*M* (monomer) with 2 m*M* ADP and 5 m*M* MgCl_2_. Purified *Vc*DciA was added to a final concentration of 0.138 m*M* (about seven monomers of DciA per helicase hexamer) before a second step of incubation. Native protein crystals were grown in sitting drops by mixing the protein solution with the reservoir solution in a 1:1 ratio. Rhombohedral crystals of the *Vc*DnaB·*Vc*DciA·ADP:Mg^2+^ complex appeared after five days at 18°C in 0.1 *M* sodium acetate pH 4.8–5.6, 0.7–0.9 *M* potassium/sodium tartrate. For derivatization, single crystals were then soaked for 2 h at 18°C in a solution containing 1 m*M* (Ta_6_Br_12_)^2+^ cluster (JBS Tantalum Cluster Derivatization Kit from Jena Bioscience GmbH, Jena, Germany). Native crystals cryoprotected with 25% glycerol or derivative crystals cryoprotected with a 50/50 Paratone/paraffin oil mixture were flash-cooled in liquid nitrogen.

Diffraction data-collection, phasing and refinement statistics are given in Table 2 ▸. Native and derivative crystallographic data were collected on the PROXIMA-2A and PROXIMA-1 beamlines, respectively, at the SOLEIL synchrotron, Saint-Aubin, France and were processed with *XDS* (Kabsch, 2010 ▸) through *XDSME* (Legrand, 2017 ▸). The strong diffraction anisotropy was corrected using the *STARANISO* server (https://staraniso.globalphasing.org; Tickle *et al.*, 2018 ▸). The crystal structure of the *Vc*DnaB·*Vc*DciA·ADP:Mg^2+^ complex was solved by molecular replacement (MR) with *MOLREP* (Vagin & Teplyakov, 2010 ▸) using the X-ray structures of the isolated NTD (22–175) and CTD (200–461) of *Vc*DnaB·GDP:AlF_4_:Mg^2+^ as search models (PDB entry 6t66; Marsin *et al.*, 2021 ▸). Two copies of each domain were correctly positioned. The initial model was then manually corrected and completed using *Coot* (Emsley *et al.*, 2010 ▸). Significant extra electron density allowed the manual building of the isolated CTDs of two *Vc*DciA monomers, the chains of which could be assigned using the 3D model of full-length *Vc*DciA predicted by *AlphaFold*2 (Jumper *et al.*, 2021 ▸) through the *ColabFold* server (Mirdita *et al.*, 2022 ▸). Additional electron density allowed the manual positioning of the isolated NTDs of two *Vc*DciA monomers using the NMR structure of *Vc*DciA^(1–111)^ solved by Marsin *et al.* (2021 ▸) (BMRB ID 27689). Finally, manual building of the linker regions connecting the NTDs and CTDs of *Vc*DciA revealed domain swapping between symmetry-related molecules of *Vc*DciA. The structure of the *Vc*DnaB·*Vc*DciA·ADP:Mg^2+^ complex was iteratively improved by manual building steps followed by refinement cycles using native data to 2.9 Å resolution. Model refinement was conducted with *BUSTER* (Bricogne *et al.*, 2017 ▸) using 12 translation–libration–screw (TLS) motion groups, automated noncrystallographic symmetry (NCS) restraints and local structure similarity restraints (LSSR) to the target models of the *Vc*DnaB·*Vc*DciA complex predicted by *AlphaFold*2 (Mirdita *et al.*, 2022 ▸; Jumper *et al.*, 2021 ▸) and *RoseTTAFold* (Baek *et al.*, 2021 ▸).

To avoid model bias, an experimental electron-density map was obtained at 3.7 Å resolution by single-wavelength anomalous diffraction (SAD) using derivative data collected at the tantalum peak wavelength. The (Ta_6_Br_12_)^2+^ cluster sites were initially found with *SHELXD* (Schneider & Sheldrick, 2002 ▸); the phases were then determined with *Phaser* (McCoy *et al.*, 2007 ▸) and improved by density modification with *Parrot* (Cowtan, 2010 ▸) in the *CCP*4 suite (Winn *et al.*, 2011 ▸). Superimposing the MR model on the experimental map confirmed its accuracy, except for the NTD of the second *Vc*DciA monomer, for which no density was visible, likely due to too sharp solvent flattening. Crystals of the *Vc*DnaB·*Vc*DciA·ADP:Mg^2+^ complex were analyzed by SDS–PAGE and both *Vc*DnaB and *Vc*DciA were visualized on the gel after Coomassie Blue staining as full-length proteins without any proteolysis (Supplementary Fig. S1). *BUSTER* (Bricogne *et al.*, 2017 ▸) calculated per-residue values for real-space correlation of the final refined model against the 2*F*_o_ − *F*_c_ map. The NTD of the second *Vc*DciA molecule has an acceptable mean main-chain real-space correlation coefficient (RSCC) of about 0.74, although this is a little lower than the mean RSCC of about 0.84 for the NTD of the first *Vc*DciA, which is very similar to the overall average RSCC of 0.83 for the whole model. This reflects a difference in flexibility between the NTDs of the two *Vc*DciA monomers, which is likely to be due to crystal-packing and domain-swapping constraints.

The structure of the *Vc*DnaB_2_·*Vc*DciA_2_ heterotetramer forming the biological assembly can be reconstructed from the domains swapped between the symmetric *Vc*DciA molecules. The resulting unswapped model consists of one *Vc*DnaB dimer interacting with two *Vc*DciA molecules, each formed by one NTD (Met1–Pro98) and one CTD (Glu122–Asp157) from two polypeptide chains related by a true crystallographic twofold rotation axis. Nevertheless, the conformation of the flexible hinge region (Glu99–Ser121) connecting the NTD and the CTD is only putative in these two reconstructed unswapped *Vc*DciA molecules, as Pro98 and Glu99 are no longer linked in this model, and will therefore differ from that in the swapped crystal structure. Finally, the *H*32 symmetry of the crystal reconstitutes the VcDnaB_6_·VcDciA_6_ heterododecameric complex by the assembly of the heterotetramer with two neighboring symmetry mates related by a true crystallographic threefold rotation axis.

All structural figures were prepared using *PyMOL* (DeLano, 2002 ▸).

### 2.3. Protein interaction analysis by thermal shift assay and intrinsic fluorescence variation

As described in Marsin *et al.* (2021 ▸), intrinsic fluorescence changes of tryptophan (and, at a lower level, tyrosine) were recorded at 330 and 350 nm while heating the protein sample from 35 to 95°C at a rate of 3°C min^−1^. The emission profile of tryptophan is shifted to the red when it is released into the solvent during the thermal denaturing of the protein. We used Tycho analysis (Tycho NT.6, NanoTemper Technologies GmbH, Munich, Germany) to follow the interaction between *Vc*DnaB or *Ec*DnaB and *Vc*DciA, *Vc*DciA^(1–111)^, *Ec*DnaC or *Ec*DnaC^(53-end)^. Interactions were performed in 50 m*M* HEPES pH 7.5, 150 m*M* NaCl, 1 m*M* ATP, with 20 µ*M* of each protein, in 10 µl capillary tubes. Three to five replicates were obtained to increase the confidence in the results. To detect binding, we compared the 350/330 nm fluorescence ratio of the complex with the predicted ratio that would be obtained in the absence of interaction by additivity of the fluorescence of the proteins alone (Sample Brightness at 350 nm/Sample Brightness at 330 nm).

### 2.4. Measurement of protein–DNA interaction by biolayer interferometry (BLI)

BLI experiments were conducted on an Octet RED96e system (Pall ForteBio, Fremont, California, USA) using streptavidin (SA) biosensors. BLI monitors the wavelength shifts (in nanometres) resulting from changes in the optical thickness of the sensor surface during association or dissociation of the analyte. All BLI experiments were performed at 30°C while stirring at 1000 rev min^−1^. The streptavidin biosensor was hydrated in a 96-well plate containing phosphate-buffered saline (PBS; Bio-Rad) for at least 10 min before each experiment. The 3′-biotinylated oligonucleotide oso13 [50-nucleotide ssDNA at 10 n*M*; GCAGGCTCGTTACGTAGCTGTACCG(dT)_25_-biotin] was immobilized in PBS onto the surface of the SA biosensor through a cycle of baseline (120 s), loading (300 s) and baseline (120 s). Association interactions were then monitored for 300 s in wells containing 200 µl sample at 100 n*M* *Vc*DnaB or *Ec*DnaB with different ratios of the indicated loader in HNATM1 buffer (50 m*M* HEPES pH 7, 150 m*M* NaCl, 1 m*M* ATP, 0.1% Tween 20, 1 m*M* MgCl_2_). At the end of each binding step, the sensors were transferred into protein-free HNATM1 binding buffer to follow the dissociation kinetics for 600 s. The sensors can be recycled by dipping them into 0.08% SDS for 10 s. The experiments were carried out in duplicate; only one is presented.

## 3. Results and discussion

### 3.1. The crystal structure of the *Vc*DnaB·*Vc*DciA complex forms a heterotetramer with 2:2 stoichiometry

We have previously demonstrated by functional studies that DciA from *V. cholerae* increases the loading of *Vc*DnaB onto DNA, resulting in an increased unwinding activity of the helicase (Marsin *et al.*, 2021 ▸). To understand the molecular interplay between the two proteins, we determined the crystal structure of the *Vc*DnaB·*Vc*DciA·ADP:Mg^2+^ complex (deposited as PDB entry 8a3v). The structure was solved by molecular replacement (see Section 2 and Table 2 ▸) using the *Vc*DnaB·GDP:AlF_4_:Mg^2+^ crystal structure (PDB entry 6t66; Marsin *et al.*, 2021 ▸), the *Vc*DciA^(1–111)^ NMR structure (BMRB ID 27689; Marsin *et al.*, 2021 ▸) and the full-length *Vc*DciA model predicted by *AlphaFold*2 (Jumper *et al.*, 2021 ▸), and was refined to 2.9 Å resolution. The accuracy of the final model was further verified by superimposition on an experimental electron-density map obtained at 3.7 Å resolution by single-wavelength anomalous diffraction (SAD) using derivative data from a (Ta_6_Br_12_)^2+^-cluster-soaked crystal (see Section 2 and Table 2 ▸).

**Figure 1:**
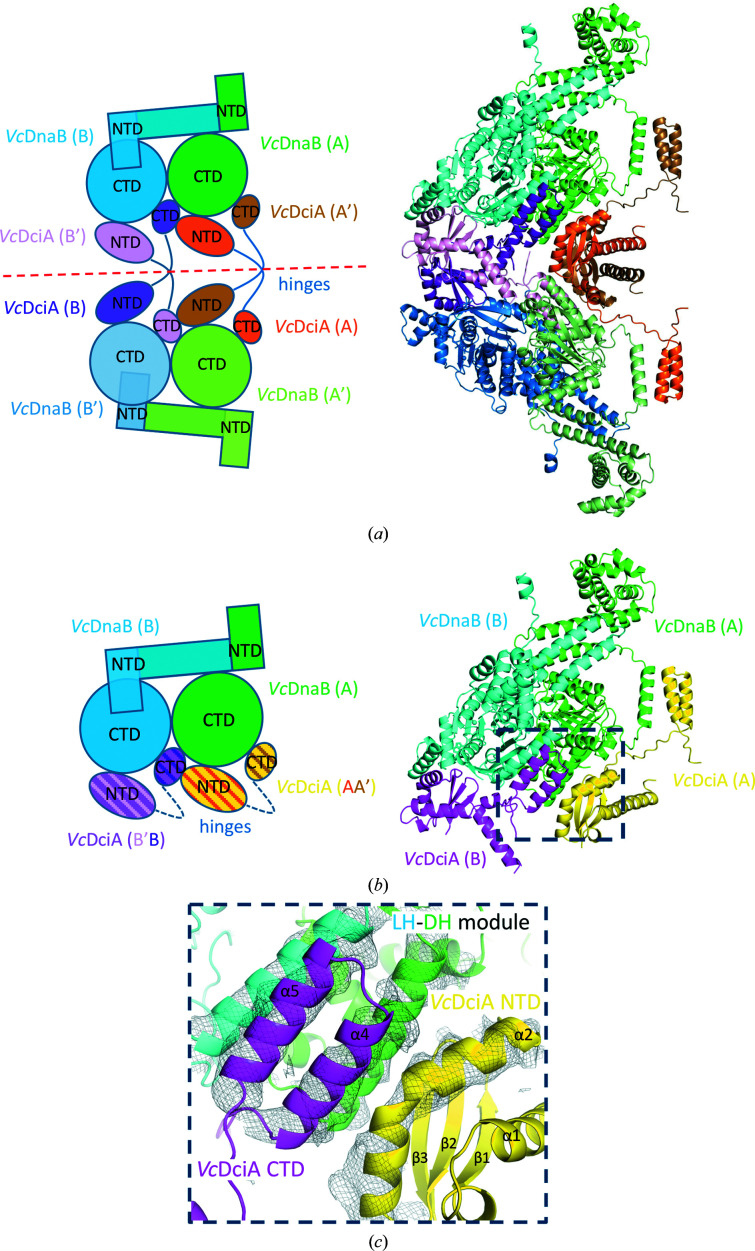
Crystal structure of the *Vc*DnaB·*Vc*DciA·ADP:Mg^2+^ complex. (*a*) Domain-swapped heterooctamer. The crystal structure of the *Vc*DnaB_2_·*Vc*DciA_2_ complex revealed domain swapping between symmetry-related molecules of *Vc*DciA. Left: schematic representation of domain swapping. The NTD and CTD of the two *Vc*DciAs, connected by an extended hinge region (dark blue lines), are exchanged between neighboring molecules related by a true crystallographic twofold rotation axis (red dashed line). The four molecules of *Vc*DnaB are in two shades of blue and green and the four molecules of *Vc*DciA are in pink, purple, orange and brown. The four protein chains of the symmetry mate are marked with a prime. Right: ribbon representation of the heterooctameric structure using the same color code. (*b*) Structure of the *Vc*DnaB·*Vc*DciA·ADP:Mg^2+^ heterotetrameric complex forming the unswapped biological assembly, reconstituted from swapped *Vc*DciA domains. Left: schematic representation. The hinges encompassing residues 99–121 of the two *Vc*DciAs are putative in this model (dark blue dotted lines). Right: ribbon representation of the heterotetrameric structure. The *Vc*DnaB dimer is in the same colors as in (*a*); the two *Vc*DciA molecules are in magenta and yellow. The dark blue dotted rectangle frames the enlarged view shown in (*c*). (*c*) Enlargement of the interface region forming a five-helix bundle, with the superimposed experimental electron-density map from SAD phasing after solvent flattening (gray mesh, contoured at 1σ).

The asymmetric unit of the crystal contains two molecules of *Vc*DnaB and two molecules of *Vc*DciA. The crystal structure of the *Vc*DnaB·*Vc*DciA complex revealed domain swapping between symmetry-related molecules of *Vc*DciA (Fig. 1 ▸ *a*). The NTD and CTD (N-terminal and C-terminal domains) of two *Vc*DciA molecules connected by an extended hinge region (residues Glu99–Ser121) are exchanged between neighboring molecules related by a true crystallographic twofold rotation axis. It is not known at present whether this domain swapping is due to a crystal artifact or whether it is biologically relevant. However, it is known that replication is bidirectional and therefore two helicases must be recruited to the replication fork (Chodavarapu & Kaguni, 2016 ▸; Hayashi *et al.*, 2020 ▸). The ability of DciA to link helicases together via this domain swapping could therefore improve the recruitment of two helicases to *oriC* and thus optimize the replication-initiation step.

The *Vc*DnaB·*Vc*DciA heterotetramer structure forming the unswapped biological assembly can be reconstructed from the domains swapped between symmetric *Vc*DciA molecules (see Section 2 and Fig. 1 ▸ *b*) and exhibits two *Vc*DciA monomers fixed on a *Vc*DnaB dimer. The *Vc*DnaB dimer in the heterotetrameric complex is bound to ADP:Mg^2+^ and is almost identical to one *Vc*DnaB dimer of the GDP-bound *Vc*DnaB hexamer structure (PDB entry 6t66; Marsin *et al.*, 2021 ▸), with an overall r.m.s.d. of 1.66 Å for 858 aligned residues (all-atom r.m.s.d.s of 1 Å for 302 aligned residues of the NTDs and 1.31 Å for 556 aligned residues of the CTDs; Supplementary Fig. S2*a* ). The NTP:Mg^2+^ binding site in the CTDs is very similar and the P-loop is in the same conformation (Supplementary Fig. S2*b* ). Therefore, formation of the complex with *Vc*DciA does not alter the overall canonical architecture of the *Vc*DnaB dimer or the NTP binding site. Nevertheless, the binding of *Vc*DciA onto the LH–DH module of *Vc*DnaB slightly accentuates the maximum gap between the C_α_ atoms of the LH and DH helices of about 5 Å relative to the module in the free *Vc*DnaB hexamer structure (Supplementary Fig. S2*a* ). The two *Vc*DciA molecules of the heterotetrameric complex are practically identical to each other (all-atom r.m.s.d.s of 0.5 Å for 78 aligned residues of the NTDs and 0.19 Å for 35 aligned residues of the CTDs), and the NTD of *Vc*DciA exhibits a KH-like fold very similar to that of the *Vc*DciA^(1–111)^ structure (BMRB ID 27689; Marsin *et al.*, 2021 ▸) that we obtained by NMR (all-atom r.m.s.d. of 1.2 Å for 78 aligned residues; Supplementary Fig. S2*c* ). However, the first long α1 helix is straight in the first *Vc*DciA molecule but is kinked with an angle of 50° at residue His24 in the second molecule (Supplementary Fig. S2*c* ). This kink can be explained because of steric hindrance with the extended hinge of the neighboring symmetric *Vc*DciA molecule with which it is engaged in domain swapping (*Vc*DciA molecules *B* and *B*′ in Fig. 1 ▸ *a*). This possibility of bending the long α1 helix of *Vc*DciA was predicted by previous molecular-dynamics analyses (Chan-Yao-Chong *et al.*, 2020 ▸). Interestingly, the CTD tail of *Vc*DciA, which was observed to be disordered in solution by SAXS (Marsin *et al.*, 2021 ▸), folds into a small helix hairpin in contact with *Vc*DnaB (Supplementary Fig. S2*d* ), again in agreement with our previous molecular-dynamics analyses (Chan-Yao-Chong *et al.*, 2020 ▸). The structure of the extended hinge region consisting of the last helix of the NTD (α3 in Supplementary Fig. S2*c* ) and the proline-rich flexible linker, which connects the NTD to the CTD of *Vc*DciA, is only putative in this unswapped biological model reconstructed from the swapped *Vc*DciA domains (see Section 2 and Fig. 1 ▸ *b*).

The two *Vc*DciA molecules interact ‘*in trans*’ with the *Vc*DnaB dimer at the periphery of its CTDs (Fig. 1 ▸ *b*). The CTD hairpin helix of one *Vc*DciA molecule stacks entirely on the LH–DH module of *Vc*DnaB (shown in magenta in Fig. 1 ▸ *c*). The kinked α2 helix of the NTD of the second *Vc*DciA (shown in yellow in Fig. 1 ▸ *c*) interacts with the first helix of the CTD hairpin of the first *Vc*DciA (approximately 238 Å^2^ ‘*in trans*’ interaction interface between the CTD and NTD from two different *Vc*DciA molecules, as measured by the *PDBePISA* server; Krissinel & Henrick, 2007 ▸) and also with the DH helix of *Vc*DnaB. The assembly forms a five-helix bundle (Fig. 1 ▸ *c*). In addition to the kinked α2 helix, the β3 strand of the *Vc*DciA NTD also interacts at the periphery of the *Vc*DnaB CTD, particularly with the DH helix. This DH helix (determinant/docking helix) is thus at the heart of the interaction between *Vc*DciA and *Vc*DnaB, as proposed by our previous Tycho experiments (Marsin *et al.*, 2021 ▸). The overall inter­action interface of a *Vc*DciA molecule with a *Vc*DnaB dimer is about 1264 Å^2^, with about 515 Å^2^ for the NTD of *Vc*DciA and about 750 Å^2^ for its CTD (as measured by the *PDBePISA* server; Krissinel & Henrick, 2007 ▸). The structure of the complex therefore confirmed the essential role played by the CTD of *Vc*DciA in the interaction with *Vc*DnaB as shown by our previous ITC experiments (Chan-Yao-Chong *et al.*, 2020 ▸). Finally, it should be noted that the NTD of the *Vc*DciA molecule with a kinked α1 helix is rather poorly defined in the density, which can be explained by the fact that its interaction interface with the CTD of *Vc*DnaB is only about 347 Å^2^, compared with 515 Å^2^ for the *Vc*DciA NTD with a straight α1 helix. This binding difference between the NTDs of the two *Vc*DciAs is likely to be caused by different steric constraints due to crystal packing and/or related to domain swapping.

### 3.2. Both the *Vc*DciA and *Ec*DnaC loaders target the conserved LH–DH module of DnaB helicases and are functionally interchangeable *in vitro*

**Figure 2:**
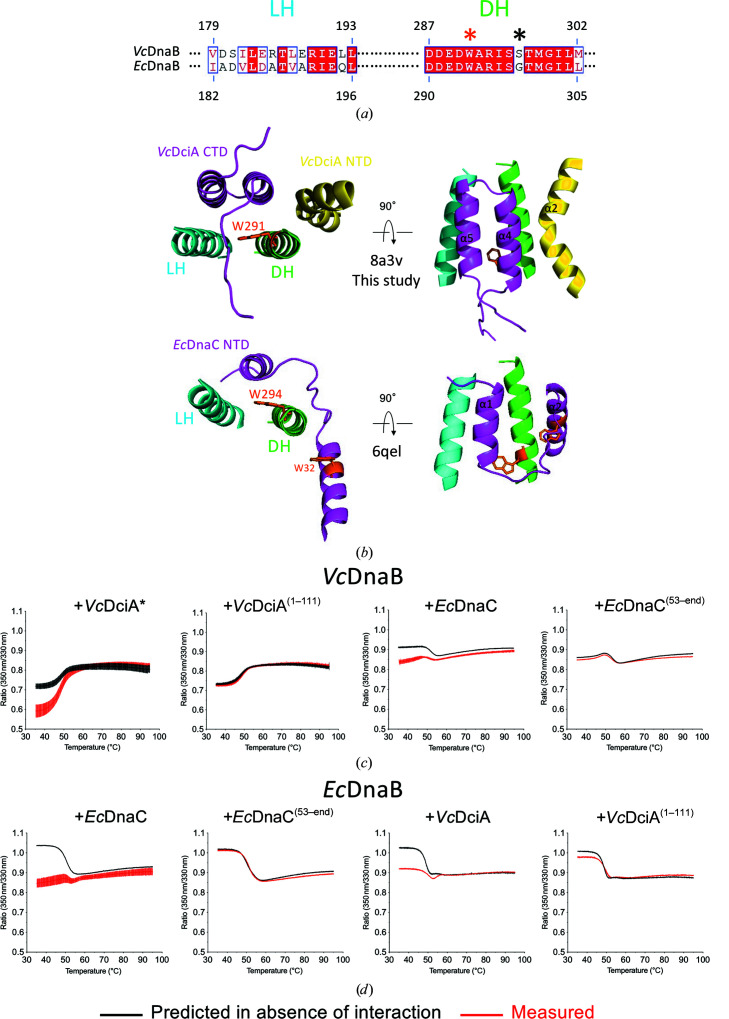
*Vc*DciA and *Ec*DnaC target the same binding site on DnaB helicases. (*a*) Sequence alignment of the LH and DH helices of *Vc*DnaB and *Ec*DnaB (generated by the EMBL–EBI *Clustal Omega* server; https://www.ebi.ac.uk/Tools/msa/clustalo/; Sievers *et al.*, 2011 ▸) and displayed using the *ESPript* 3.0 server (https://espript.ibcp.fr; Robert & Gouet, 2014 ▸). The conserved residues are in white on a red background. An orange asterisk marks the conserved tryptophan residues, while a black asterisk marks the specific serine/glycine residues in the DH helix. (*b*) Close-up view of the interaction interface forming a three- or five-helix bundle between the two-helix LH–DH module of DnaB (blue and green) and *Vc*DciA (top; magenta and yellow; PDB entry 8a3v; this study) or *Ec*DnaC NTD (bottom; magenta; PDB entry 6qel), respectively. The tryptophans whose intrinsic fluorescence variation was measured in the Tycho experiments are shown in orange sticks. (*c*, *d*) Tycho NT.6 analysis. The emission profile of a tryptophan is shifted to the red when it is released to the solvent during thermal denaturing of the protein. The 350/330 nm ratios measured for the different helicase-loader mixtures are reported in red and the predicted ratio in the absence of interaction is reported in black. The ratio comparisons are reported for each helicase-loader couple indicated, namely *Vc*DnaB (*c*) or *Ec*DnaB (*d*) with two constructs of *Vc*DciA or *Ec*DnaC. The curves correspond to the mean ± SEM of three to five analyses. The Tycho interaction analysis between *Vc*DnaB and *Vc*DciA has previously been published (Marsin *et al.*, 2021 ▸) but is reproduced here (indicated by *) for easy evaluation with other helicase-loader couples.

The LH–DH module is conserved in DnaB helicases (Chase *et al.*, 2022 ▸). However, we have previously identified a residue in the DH helix that discriminates DciA helicases from DnaC/I helicases (a serine and a glycine, respectively; Marsin *et al.*, 2021 ▸; Fig. 2 ▸ *a*). Moreover, the *Vc*DciA and *Ec*DnaC loaders have no sequence or structural similarity. Yet, the two loaders target the same binding site on the helicase: the conserved LH–DH module of DnaB, albeit with differences in the interaction interfaces (Fig. 2 ▸ *b*). This interaction involves a single small α-helix in the NTD of *Ec*DnaC, which forms a three-helix bundle, whereas in *Vc*DciA the CTD helix hairpin and the elbow of the kinked α2 helix of NTD both participate in forming a five-helix bundle. We may wonder at this stage whether this common target on DnaB, the LH–DH module, allows cross-talk between the two helicase-loader systems, despite their specificities.

Using Tycho nano-DSF technology, the fluorescence variation of tryptophan residues can be followed in real time along a thermal ramp (see Section 2). Ideally, a conserved tryptophan is located in the DH helix, namely Trp291 in *Vc*DnaB and Trp294 in *Ec*DnaB (Figs. 2 ▸ *a* and 2 ▸ *b*; Marsin *et al.*, 2021 ▸). A second conserved tryptophan is located in the globular head of the NTD domain of DnaB (positions 45 and 48 in *Vc*DnaB and *Ec*DnaB, respectively), but is buried and cannot participate in any protein–protein interactions. *Vc*DciA does not contain any tryptophans in its sequence. We have previously demonstrated the binding of *Vc*DciA in the proximity of the LH–DH module of *Vc*DnaB using this technology (Marsin *et al.*, 2021 ▸), showing that Trp291 is in­accessible to solvent when *Vc*DnaB interacts with *Vc*DciA. We further showed that *Vc*DciA with its CTD deleted [*Vc*DciA^(1–111)^] can no longer bind *Vc*DnaB (Fig. 2 ▸ *c*, left), as would be expected if the CTD of *Vc*DciA covers the DH α-helix of *Vc*DnaB (Fig. 2 ▸ *b*). On the other hand, *Ec*DnaB contains a third nonconserved tryptophan residue (Trp457) that is solvent-exposed in its CTD and is at the interface with the loader in the structure of the *Ec*DnaB·*Ec*DnaC complex (PDB entry 6qel; Arias-Palomo *et al.*, 2019 ▸). *Ec*DnaC also encloses three solvent-exposed tryptophan residues in its sequence, with one in its NTD extended end at the interface with the helicase in the structure of the *Ec*DnaB·*Ec*DnaC complex (Trp32 in Fig. 2 ▸ *b*; PDB entry 6qel; Arias-Palomo *et al.*, 2019 ▸) and two in its CTD. All of these tryptophan residues could therefore participate in protein–protein interactions. Indeed, Tycho allowed confirmation of the interaction between *Ec*DnaB and *Ec*DnaC by showing a lower initial 350/330 nm ratio for the measured curve (in red; Fig. 2 ▸ *d*, left) compared with the theoretical curve representing the absence of solvent protection (in black; Fig. 2 ▸ *d*, left). In addition, *Ec*DnaC with its NTD deleted [*Ec*DnaC^(53-end)^] can no longer bind *Ec*DnaB (Fig. 2 ▸ *d*, left), as would be expected if the α1 helix of *Ec*DnaC forms a three-helix bundle with the LH–DH module of *Ec*DnaB (Fig. 2 ▸ *b*). These findings are in agreement with the 3D structures of the two complexes: the interaction takes place at the DH of DnaB and requires the CTD for DciA and the NTD for DnaC. We further carried out crossover experiments using the heterologous *Vc*DnaB/*Ec*DnaC and *Ec*DnaB/*Vc*DciA systems. We showed efficient noncognate helicase-loader interactions, which also require the NTD of *Ec*DnaC (Fig. 2 ▸ *c*, right) and the CTD of *Vc*DciA (Fig. 2 ▸ *d*, right).

**Figure 3:**
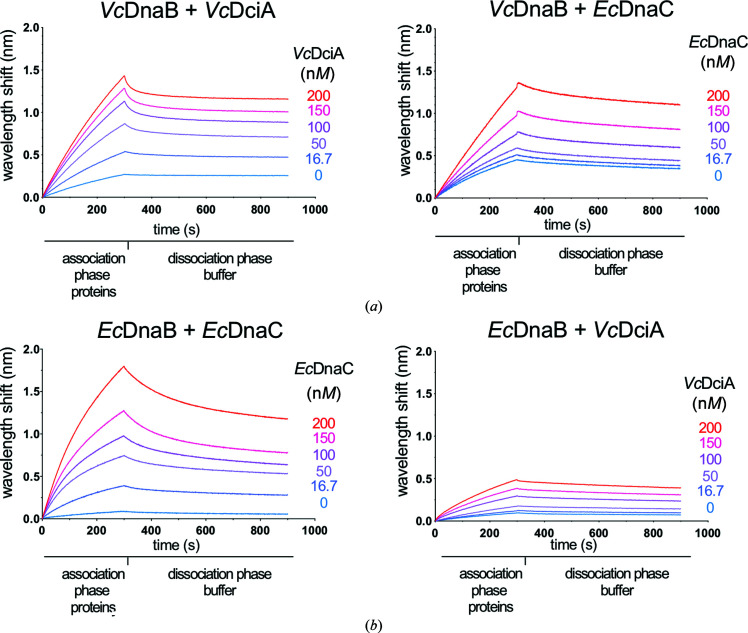
*Vc*DciA and *Ec*DnaC loaders are functionally interchangeable *in vitro*. Biolayer interferometry (BLI) analysis using a biotinylated oligonucleotide (50 nucleotides) immobilized onto the surface of an SA-coated probe by its 3′ extremity (see Section 2). Association was performed with the indicated helicase at a concentration of 100 n*M* during 300 s in a buffer solution containing ATP and MgCl_2_. Dissociation was assessed in the same buffer for 600 s. Increasing loader concentrations (from 0 to 200 n*M* in subunits; blue to red) were analyzed. The experiments were carried out in duplicate; only one is presented. (*a*) *Vc*DnaB binding on ssDNA in the presence of *Vc*DciA (left) or *Ec*DnaC (right). (*b*) *Ec*DnaB binding on ssDNA in the presence of *Ec*DnaC (left) or *Vc*DciA (right).

We then investigated whether these interactions are relevant for the stimulation of helicase loading onto DNA by the loaders. We attached a 3′-biotinylated 50-mer ssDNA to a streptavidin-coated probe to measure interactions using biolayer interferometry (BLI; see Section 2). We monitored interactions between immobilized ssDNA and the helicases in real time in the presence of different concentrations of loaders (Fig. 3 ▸). The BLI experiments confirmed the results previously observed by SPR (Marsin *et al.*, 2021 ▸), namely that the loading stimulation of both helicases increases with the concentration of added cognate loader, *Vc*DciA or *Ec*DnaC (Figs. 3 ▸ *a* and 3 ▸ *b*, left), at concentrations for which the response for loaders alone is negligible (Supplementary Fig. S3). Moreover, under the same conditions *Ec*DnaC is able to efficiently load *Vc*DnaB onto the ssDNA, and *Vc*DciA is able to load *Ec*DnaB, although less effectively than *Ec*DnaC (Figs. 3 ▸ *a* and 3 ▸ *b*, right). The cross-talk is verified *in vitro* and there seems to be a functional convergence between the two systems. This could explain why the replacement of DciA by DnaC/I has occurred at least seven times during evolution, and how phage loaders have been able to hijack bacterial replicative helicases efficiently (Brézellec *et al.*, 2016 ▸).

### 3.3. *Vc*DciA binds to the periphery of the *Vc*DnaB CTD, in contrast to other loaders, which are positioned at the back of the helicase CTD ring

**Figure 4:**
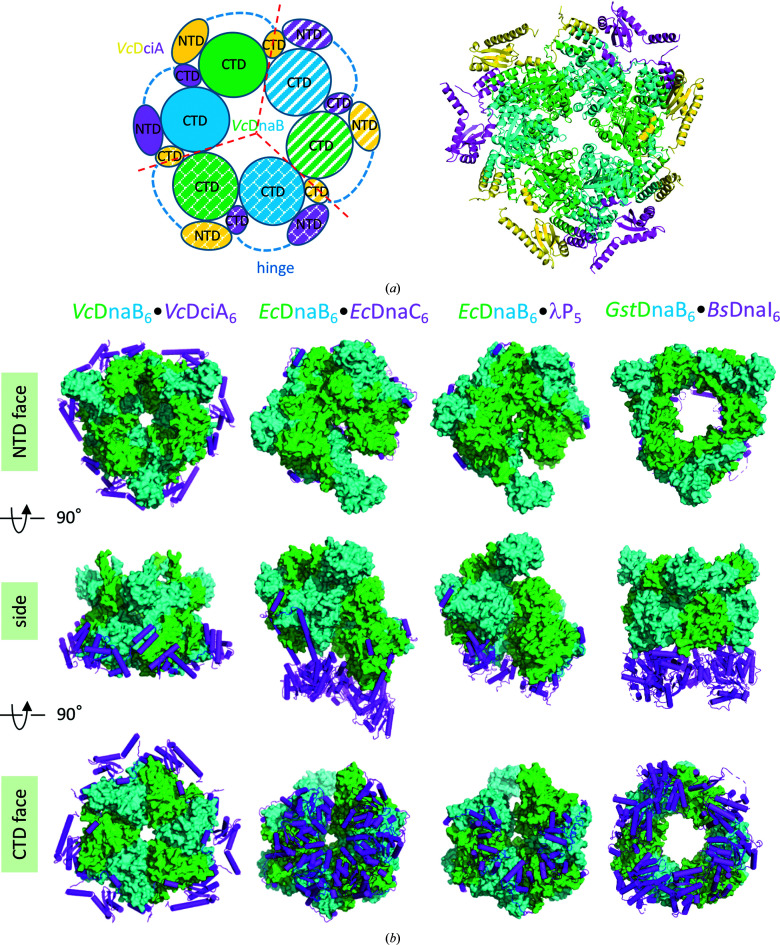
DciA binds to the periphery of the DnaB CTD. (*a*) Structure of the *Vc*DnaB_6_·*Vc*DciA_6_ heterododecameric complex reconstituted by the crystal *H*32 symmetry. The color code is the same as in Fig. 1 ▸(*b*). Left: schematic representation. The heterododecameric ring is reconstituted by assembly of the heterotetramer with two neighboring symmetry mates (hatched textures) related by a true crystallographic threefold rotation axis (red dashed lines). The hinges encompassing residues 99–121 of the *Vc*DciA molecules are putative in this unswapped model (dark blue dotted lines). Right: ribbon representation of the heterododecameric model. (*b*) Structural comparison of four helicase-loader complexes: *Vc*DnaB·*Vc*DciA (PDB entry 8a3v, this study), *Ec*DnaB·*Ec*DnaC (PDB entry 6qel), *Ec*DnaB·λP (PDB entry 6bbm) and *Gst*DnaB·*Bs*DnaI (extracted from PDB entry 4m4w). The DnaB hexamers are represented as surfaces (blue and green) and the helicase loaders as magenta sticks. Unlike DnaC, λP and DnaI, which cover the back of the DnaB CTD ring, DciA leaves it free by positioning itself at the periphery of the helicase.

Our previous SEC–SAXS and SEC–MALS experiments showed that a complex between the *Vc*DnaB hexamer and *Vc*DciA is formed in solution under specific *in vitro* conditions with a predominant 6:3 stoichiometry (Marsin *et al.*, 2021 ▸). However, this complex is not stable and tends to dissociate, showing that it is dynamic in solution, probably due to rapid molecular exchanges leading to a mixture of several conformational states (Marsin *et al.*, 2021 ▸). In the current study, the experimental conditions were optimized to compare the *V. cholerae* and *E. coli* helicase-loader systems and the BLI curves did not reach a saturation plateau even at a ratio of two *Vc*DciA molecules to one *Vc*DnaB molecule (Fig. 3 ▸ *a*, left). Interestingly, the *H*32 symmetry of the crystal reconstitutes a *Vc*DnaB_6_·*Vc*DciA_6_ heterododecamer complex by the assembly of the heterotetramer with two neighboring symmetry mates related by a true crystallographic threefold rotation axis (Fig. 4 ▸ *a*). Of course, a crystallographic structure captures only one of the possible conformational intermediates, with a 2:2 or 6:6 stoichiometry in our case, and it is not certain that the current structure is that of an active state. Further work remains to be performed to determine whether or not this heterododecameric structure is biologically relevant, but in the meantime we can compare it with other helicase-loader structures in the literature. Three 3D structures of helicase-loader complexes are currently known (see Table 1 ▸): *Ec*DnaB·*Ec*DnaC (PDB entry 6qel; Arias-Palomo *et al.*, 2019 ▸), *Ec*DnaB·λP (PDB entry 6bbm; Chase *et al.*, 2018 ▸) and *Gst*DnaB·*Bs*DnaI (PDB entry 4m4w; Liu *et al.*, 2013 ▸). Strikingly, *Vc*DciA binds to the periphery of the *Vc*DnaB CTD, in contrast to the other three loaders, which oligomerize and are positioned at the back of the helicase CTD ring (Fig. 4 ▸ *b*). This discrepancy leads us to consider that the loading mechanism used by DciA, which has still to be elucidated, could differ from those previously described for the other three loaders. However, it is possible that a partner, a protein or a nucleic acid, remains to be discovered in order to fully decipher the function of DciA.

## 4. Conclusions

The genes coding for DciA and DnaC/I are unrelated and are mutually exclusive in bacterial genomes (Brézellec *et al.*, 2017 ▸; Brézellec *et al.*, 2016 ▸). However, like DnaC and the bacteriophage λ P helicase loader (Chase *et al.*, 2018 ▸, 2022 ▸; Arias-Palomo *et al.*, 2019 ▸), DciA interacts with the two-helix LH–DH module of DnaB. It is not yet known whether DnaI also targets the LH–DH module of the replicative helicase. In the available low-resolution crystal structure of the *Gst*DnaB·*Bs*DnaI complex (PDB entry 4m4w; Liu *et al.*, 2013 ▸), several parts of the NTD end of DnaI, which have already been identified to interact with DnaB (Loscha *et al.*, 2009 ▸; Tsai *et al.*, 2009 ▸), as well as the LH of the DnaB module (which is also conserved in *Gst*DnaB; PDB entries 2r6a and 4esv; Bailey *et al.*, 2007 ▸; Itsathitphaisarn *et al.*, 2012 ▸), are not visible, preventing us from definitively concluding that there is a common binding site for the various helicase loaders. However, *AlphaFold-Multimer* (Evans *et al.*, 2022 ▸; Mirdita *et al.*, 2022 ▸) predicts an interaction between the NTD of DnaI and the LH–DH module of DnaB (Supplementary Fig. S4), making a universal site of interaction plausible. This ‘binding module’, which is conserved in the bacterial replicative helicases, as well as the fact that cross-talk reactions are efficient *in vitro* between the DciA and DnaC systems, suggest convergent evolution of the different helicase-loader systems. Nevertheless, the structural data on the *Vc*DnaB·*Vc*DciA·ADP:Mg^2+^ complex provided here do not permit us to postulate that the loading mechanism used by DciA will be of the ‘ring-breaker’ or ‘ring-maker’ type, or even of a third type which remains to be elucidated. This conformational state may be an inactive intermediate state before its activation by a third partner that has yet to be discovered. A recent computational evolutionary study showed that DciA homologs exhibit a tremendous diversity of domain architectures across bacterial phyla (Blaine *et al.*, 2022 ▸). Notably, one group of DciA homologs only encodes a KH-like fold and no other N- or C-terminal extensions, suggesting that an additional partner may indeed be required for DciA function. Thus, future investigations will probably uncover a cofactor, a protein or a nucleic acid, that is necessary for DciA to complete its helicase-loading cycle.

## Supplementary Material

PDB reference: *Vc*DnaB·*Vc*DciA, 8a3v

Supplementary Figures. DOI: 10.1107/S2059798323000281/jb5052sup1.pdf

## Figures and Tables

**Table 1:** Structural states of the DnaB hexamer in complex with various helicase loaders and their loading mechanisms

Complex	Stoichiometry	Origin of the helicase	Origin of the loader	PDB code	Resolution (Å)	Helicase ring state	Loading mechanism
DnaB·DnaI·DnaG	6:6:3	*Geobacillus stearothermophilus*	*Bacillus subtilis*	4m4w	6.10	Closed and dilated planar	Ring-maker
DnaB·DnaC	6:6	*Escherichia coli*	*Escherichia coli*	6qel	3.90	Open helical	Ring-breaker
DnaB·λP	6:5	*Escherichia coli*	Phage λ	6bbm	4.10	Open helical	Ring-breaker

**Table 2:** Data-collection, phasing and refinement statistics for *Vc*DnaB·*Vc*DciA·ADP:Mg^2+^ Values in parentheses are for the highest resolution shell.

	Native†	(Ta_6_Br_12_)^2+^ derivative‡
Data collection		
Space group	*H*32	*H*32
*a*, *b*, *c* (Å)	186.51, 186.51, 252.84	186.67, 186.67, 252.99
α, β, γ (°)	90.0, 90.0, 120.0	90.0, 90.0, 120.0
Wavelength (Å)	0.984	1.254
Resolution range (Å)	48.3–2.9 (3.1–2.9)	49.8–3.7 (3.8–3.7)
Before *STARANISO*		
Measured/unique reflections	651973/37637	423037/18409
Spherical completeness (%)	99.9 (99.9)	99.1 (94.5)
Spherical anomalous completeness (%)		98.6 (86.4)
〈*I*/σ(*I*)〉	7.9 (0.3)	6.0 (0.5)
After *STARANISO*		
Measured/unique reflections	368086/21186	259130/11065
Ellipsoidal completeness (%)	94.1 (71.7)	95.6 (97.0)
Ellipsoidal anomalous completeness (%)		95.6 (94.7)
〈*I*/σ(*I*)〉	13.4 (1.6)	12.6 (2.0)
*R*_merge_ (%)	19.6 (236.0)	22.3 (213.9)
*R*_p.i.m._ (%)	4.6 (54.1)	4.7 (45.6)
Multiplicity	17.4 (19.9)	23.4 (22.3)
Anomalous multiplicity		12.2 (11.6)
CC_1/2_	0.999 (0.544)	0.999 (0.671)
CC_ano_		0.899 (0.0)
|DANO|/σ(DANO)		1.588 (0.767)
SAD phasing		
No. of sites		7
Overall FOM		0.311
Overall FOM after density modification		0.693
Refinement		
Resolution range (Å)	40.8–2.9	
No. of work/test reflections	20312/1229	
*R*/*R*_free_ (%)	27.9/29.0	
Geometry statistics		
No. of atoms		
Total	9367	
Protein	9311	
Ligand/ion	56	
Water	0	
R.m.s.d. from ideal values		
Bond lengths (Å)	0.005	
Bond angles (°)	0.72	
Average *B* factors (Å^2^)		
Overall	125.3	
Protein	125.2	
Ligand/ion	131.4	
Ramachandran plot		
Most favored (%)	96.6	
Outliers (%)	0.5	
*MolProbity* score	2.05	

† Diffraction data were collected from one crystal which diffracted anisotropically to 2.88 Å resolution along 0.894a* − 0.447b*, to 2.88 Å resolution along b* and to 5.21 Å resolution along c*.

‡ Diffraction data collected were from one crystal which diffracted anisotropically to 3.43 Å resolution along 0.894a* − 0.447b*, to 3.43 Å resolution along b* and to 6.50 Å resolution along c*.
