# Supplementary material for: The LH–DH module of bacterial replicative helicases is the common binding site for DciA and other helicase loaders
Source: Acta Crystallogr D Struct Biol. 2023 Feb 6;79(Pt 2):177–87. doi: 10.1107/S2059798323000281 (PMC9912922; doi:10.1107/S2059798323000281)
Supplement: Supplementary file 1 [file d-79-00177-sup1.pdf]

Acta Cryst D

STRUCTURAL BIOLOGY

Volume 79 (2023)

Supporting information for article:

The LH-DH module of the bacterial replicative helicases is the common binding site for DciA and other helicase loaders

Claire Cargemel, Stéphanie Marsin, Magali Noiray, Pierre Legrand, Halil Bounoua, Inès Li de la Sierra-Gallay, Hélène Walbott and Sophie Quevillon-Cheruel

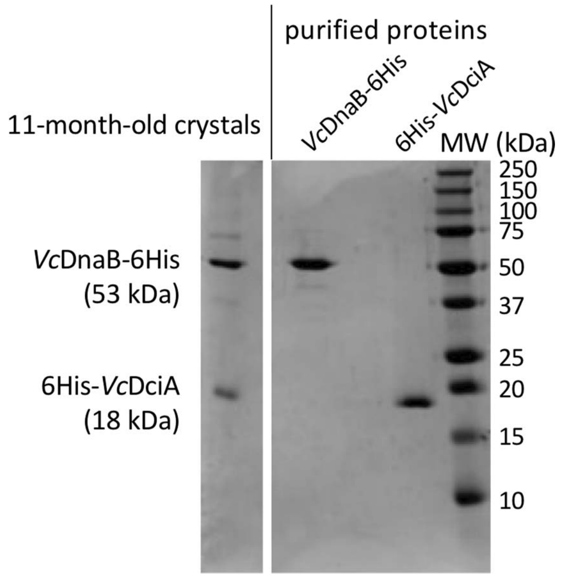

Figure S1 Validation of the protein composition of the *Vc*DnaB•*Vc*DciA complex crystals. 11-month-old crystals of the complex were visualized by SDS-PAGE and Coomassie Blue staining. Purified *Vc*DnaB and *Vc*DciA proteins were migrated on the same gel as controls, showing that the crystallized proteins were protected from proteolysis. The crystals of the complex obtained in our study grew in 5 days and were formed from the two full-length proteins.

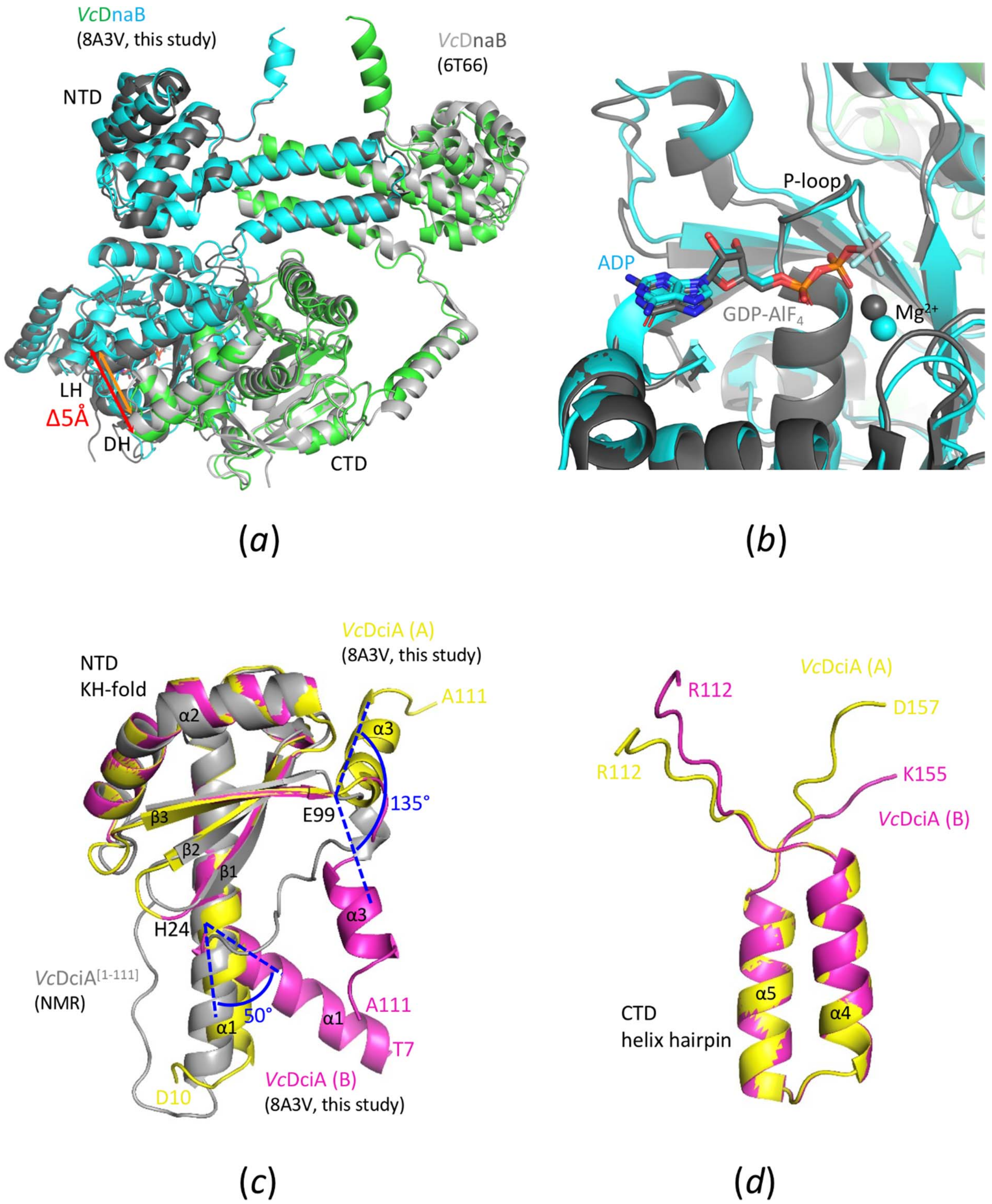

Figure S2 Interaction with *Vc*DciA does not alter the overall structure of the *Vc*DnaB dimer but induces folding of the *Vc*DciA CTD into a helix hairpin. ***(a)*** Superimposition of the ADP-bound *Vc*DnaB dimer extracted from the complex with *Vc*DciA (cyan and green, PDB ID 8A3V, this study) with a GDP-bound *Vc*DnaB dimer extracted from the hexameric helicase ring (gray, PDB ID 6T66). The two structures of *Vc*DnaB are almost identical (global RMSD of 1.66 Å for 858 aligned residues). However, the maximum distance between the Cα atoms of the LH and DH helices is increased by about 5 Å in the presence of *Vc*DciA (compare double red arrow with double orange arrow). ***(b)***

Zoom-in on the NTP binding site of the two superimposed *Vc*DnaB dimer structures (same colours as in (*a*)). The P-loops are perfectly superimposable, as well as the ADP (blue sticks) and the GDP-AlF_4 (gray sticks), and also the Mg^{2+} ions (cyan and gray spheres). ***(c)*** Superimposition of the two *Vc*DciA NTDs (yellow and magenta, PDB ID 8A3V, this study) extracted from the *Vc*DnaB•*Vc*DciA complex with the NMR structure of the isolated NTD of *Vc*DciA (in gray, BMRB ID 27689). The KH-like fold of the *Vc*DciA NTDs in the complex is very similar to the *Vc*DciA$^{[1\text{-}111]}$ NMR solution structure (all-atom RMSD of 1.2 Å for 78 aligned residues). The two copies of *Vc*DciA NTD in the heterotetrameric structure are identical (all-atom RMSD of 0.5 Å for 78 aligned residues), except for the first long α1 helix (bend at residue H24 in one copy, with an angle of 50° as illustrated by blue lines) and the last α3 helix (oriented almost oppositely in the two copies with an angle of 135° as illustrated by blue lines). This reflects a certain flexibility of *Vc*DciA which was predicted by previous molecular dynamics analyses (Marsin *et al.*, 2021). ***(d)*** Superimposition of the two *Vc*DciA CTDs (same colours as in (*c*)) extracted from the complex with *Vc*DnaB. The *Vc*DciA CTD, which was shown disordered in solution by SAXS (Marsin *et al.*, 2021), folds into a helix hairpin upon interaction with the *Vc*DnaB dimer (contact with the LH-DH module of the helicase). The two copies of *Vc*DciA CTD are identical (all-atom RMSD of 0.19 Å for 35 aligned residues).

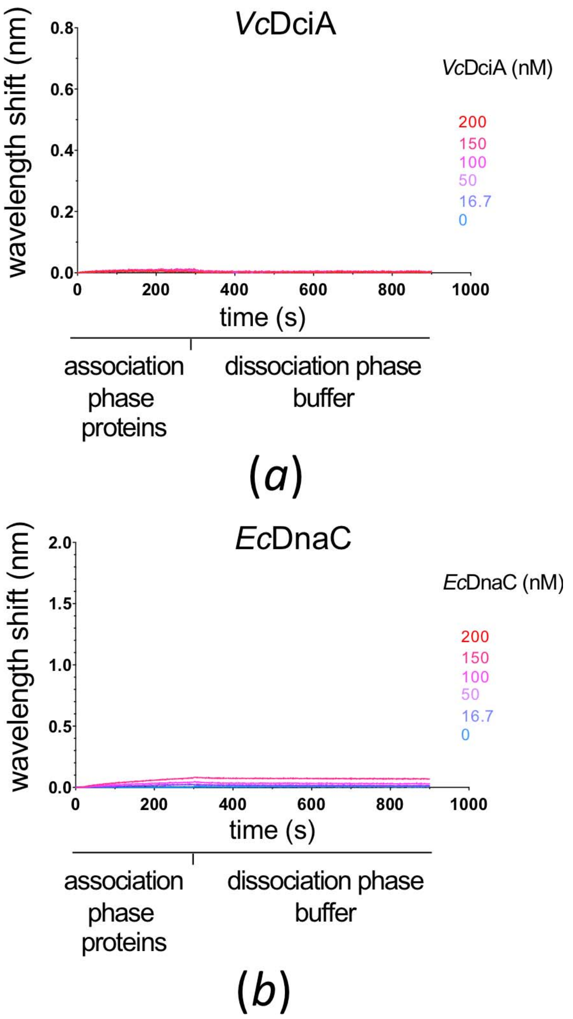

Figure S3 Interaction with ssDNA is detectable for neither ***(a)*** *Vc*DciA nor ***(b)*** *Ec*DnaC under the same experimental conditions used for the helicase loading assays in this study. The Bio-layer interferometry (BLI) analysis of the two loaders alone (from 0 to 200 nM in subunits, in blue to red), is performed as described in Fig. 3 and in the Materials and methods section.

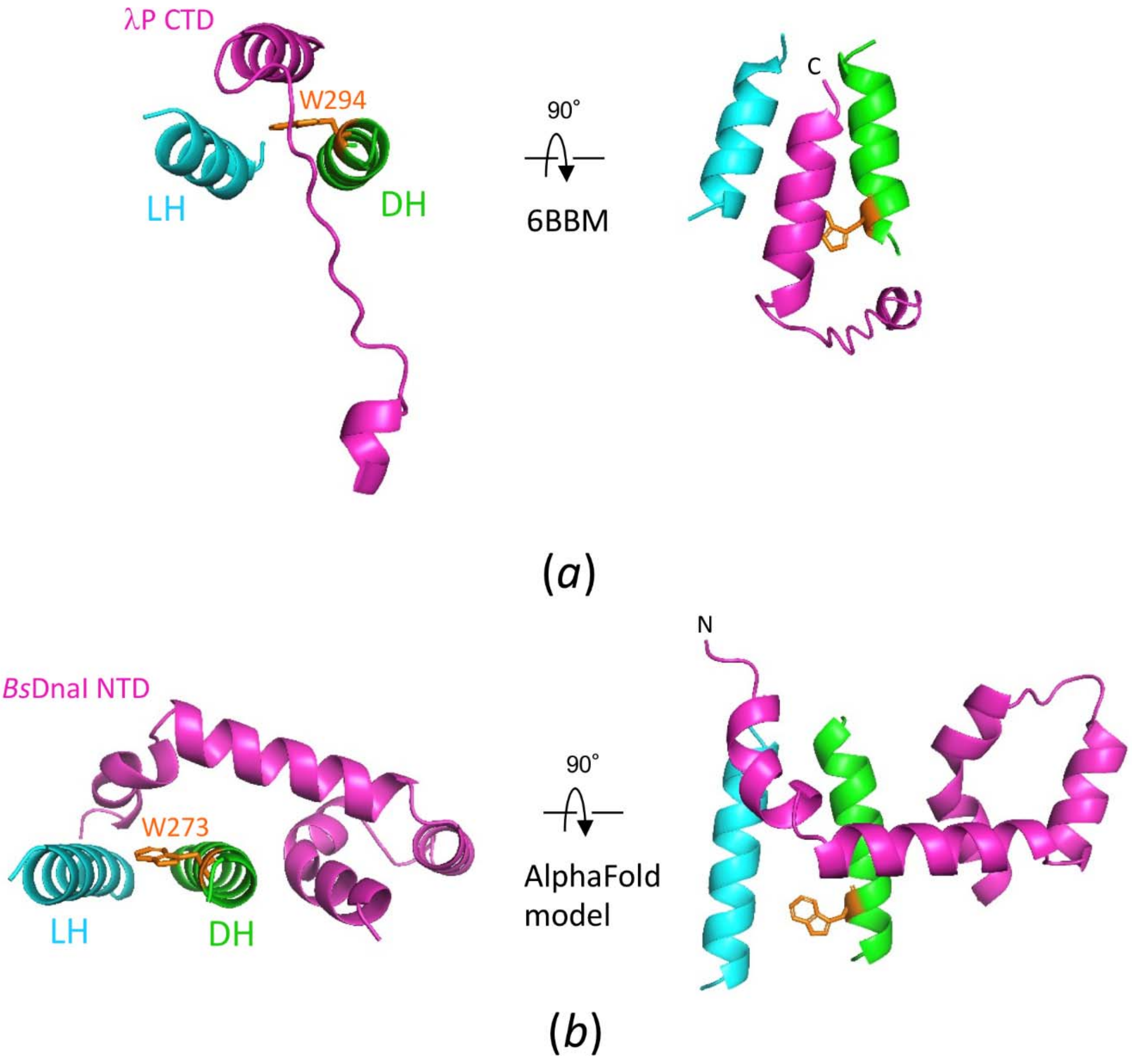

Figure S4 The LH-DH module of bacterial replicative helicases is the common binding site for unrelated helicase loaders. **(*a*)** Zoom-in of the interaction interface between the two-helix LH-DH module of *Ec*DnaB (in blue and green) and λP CTD (in magenta, PDB ID 6BBM). **(*b*)** Zoom-in of the interaction interface between the LH-DH module of *Gst*DnaB (in blue and green) and *Bs*DnaI NTD (in magenta, model of the *Gst*DnaB•*Bs*DnaI complex generated by AlphaFold-Multimer (Mirdita *et al.*, 2022, Evans *et al.*, 2022). The close-up orthogonal views and the color code are the same as in Fig. 2*b*. The conserved tryptophans in the DH are in orange sticks.
